# Fungi associated with the potato taste defect in coffee beans from Rwanda

**DOI:** 10.1186/s40529-022-00346-9

**Published:** 2022-05-23

**Authors:** Amanda R. Hale, Paul M. Ruegger, Philippe Rolshausen, James Borneman, Jiue-in Yang

**Affiliations:** 1 Department of Microbiology and Plant Pathology, University of California, Riverside, CA 92521 USA; 2 Department of Botany and Plant Sciences, University of California, Riverside, CA 92521 USA; 3 Department of Plant Pathology and Microbiology, National Taiwan University, Taipei, 10617 Taiwan

**Keywords:** High-throughput sequencing, rRNA ITS region, Amplicon sequencing, Microbiome

## Abstract

**Background:**

Potato taste defect (PTD) of coffee is characterized by a raw potato like smell that leads to a lower quality taste in the brewed coffee, and harms the commercial value of some East African coffees. Although several causes for PTD have been proposed, none of them have been confirmed. Recently, high throughput sequencing techniques and bioinformatic analysis have shown great potential for identifying putative causal agents of plant diseases. Toward the goal of determining the cause of PTD, we examined raw coffee beans from Rwanda exhibiting varying PTD scores using an Illumina-based sequence analysis of the fungal rRNA ITS region.

**Results:**

Six fungal amplicon sequence variants (ASVs) with high relative abundances correlated with coffee taste scores. Four of these ASVs exhibited negative correlations – *Aspergillus versicolor, Penicillium cinnamopurpureum, Talaromyces radicus,* and *Thermomyces lanuginosus* – indicating that they might be causing PTD. Two of these fungi exhibited positive correlations – *Kazachstania humilis* and *Clavispora lusitaniae* – indicating that they might be inhibiting organisms that cause PTD.

**Conclusions:**

This study addressed PTD causality from a new angle by examining fungi with high throughput sequencing. To our knowledge, this is the first study characterizing fungi associated with PTD, providing candidates for both causality and biocontrol.

## Background

Coffee is the most valuable traded beverage, and the second most widely distributed commodity after crude oil, with an estimated annual trade value exceeding 30 billion US dollars in 2015 (Torok et al. 2018). Many East African countries suffer from the “potato taste defect” (PTD), which creates an unpalatable potato flavor in the brewed coffee (Jackels et al. 2014). Since coffee field crop examiners and bean buyers don’t have effective methods to identify the affected beans, the problem continues, increasing costs and decreasing profits (Jackels et al. 2014).

There is no evidence linking a specific post-harvest preparation method to PTD. Coffee berries are immediately processed after harvest, with either the dry or wet method (Hamdouche et al. 2016). The dry method involves sorting, sun-drying, and then husking the coffee berries, while the wet method first ferments the coffee in water before drying and husking (Hamdouche et al. 2016). Both methods are used in the PTD-afflicted East Africa region. For example, Rwanda and Burundi use the wet method whereas Congo uses the dry method (Mutua 2000).

Specific chemicals in coffee beans have been linked to PTD, including several that are commonly produced by bacteria and fungi. Becker et al. (1987) showed that the metabolite 2-isopropyl-3-methoxypyrazine (IMP) was 30 times higher in PTD-affected green coffee beans from Rwanda, and it has an odor similar to the one emanating from brewed coffee with PTD. These authors suggested that IMP is formed during the growth or preparation of the coffee beans, and not during the roasting process. Another compound detected in raw and roasted Arabica coffee beans, 3-isobutyl-2-methoxypyrazine, was determined to be the major contributing pyrazine linked to PTD, among other odorants (Czerny and Grosch 2000). In addition, tridecane, dodecane, and tetradecane were more abundant on the surface of PTD-affected coffee beans compared to control beans (Jackels et al. 2014). Mold fungi are common culprits of foul tastes and odors, and they produce a wide variety of metabolites including IMP (Bennett and Inamdar 2015). Bacteria are also known to produce potato-like odors due to alkyl-methoxypyrazines. For example, one study showed that *Pseudomonas perolens* produces 2-methoxy-3-isopropylpyrazine (Cheng et al. 1991)*.* Another study found that *Serratia odorifera, Serratia ficaria*, *Serratia rubidaea*, and *Cedecea davisae* produced the odor-associated metabolites 2-methoxy-3-isopropylpyrazine, 3-isopropyl-2-methoxy-5-methyl-pyrazine, and 3-s-butyl-2-methoxypyrazine, among others (Gallois and Grimont 1985). Finally, the bacterium *Pantoea coffeiphila* sp. nov. has been proposed to cause PTD, because it was found in PTD-affected coffee beans, and there is a link between this bacterium and IMP (Gueule et al. 2015).

The insect *Antestiopsis thunbergii* (the Antestia bug) has also been implicated in PTD. It is believed that when the Antestia bug physically damages coffee berries, it allows a microbe to infect the berry (Bigirimana et al. 2018). Another theory that does not include a microbial component is that the Antestia bug induces a plant stress response where the plant itself produces the PTD-associated chemicals (Jackels et al. 2014). Furthermore, surface volatiles found on PTD coffee beans (tridecane, dodecane, and tetradecane) were also found on Antestia bugs (Jackels et al. 2014). Antestia bug management techniques such as coffee plant pruning and pesticide application reduce the levels of both the Antestia bug and PTD (Bigirimana et al. 2018). In addition, Antestia bugs carry putative bacterial symbionts in their midguts and ovaries (Matsuura et al. 2014), which presents the possibility that this insect could vector PTD-causing microorganisms. Finally, the Antestia bug was discovered to be a vector of the fungal plant pathogen, *Eremothecium gossypii,* causing dry rot in the beans of *Coffea robusta* in Uganda and *Coffea arabica* in other East African countries (Pridham and Raper 1950). That study showed that *E. gossypii* only infected the beans when the fungus was injected into the coffee berry. However, at this time, no Antestia bug-associated microorganisms, or their metabolites, have been linked to PTD.

In this study, we attempt to assess PTD causality from a new angle, using an Illumina-based sequence analysis to determine the relationships between fungal rRNA ITS sequences and raw coffee beans from Rwanda exhibiting varying PTD scores. To our knowledge, this is the first study characterizing the fungi associated with PTD.

## Methods

### Coffee samples and taste scores

Coffee beans were grown and harvested on farms in the Southern Province in Rwanda, and then transported to the Rogers Family Company in Lincoln California (USA) as part of the company’s normal business operation. In 2013, subsamples of raw coffee beans from 24 commercial shipping bags were collected, and portions of each subsample were roasted for cupping evaluation. The Roger's Family Company standard procedure is to brew coffee using 8.25 g of roasted beans with 150 ml of hot water and then score the coffee for taste. Taste scores are shown in Table 1. The other portions of these subsamples (~ 100 g per subsample) were sent to our labs for microbiological analyses. Taste scores were assigned by cupping experts using the standards of the Specialty Coffee Association of America (Specialty Coffee Association 2020), which reflects the overall quality of the coffee bean by evaluating the fragrance/aroma, flavor, aftertaste, acidity, body, balance, sweetness, uniformity, and cleanliness. The scoring system used a 100-point scale, with scores ranging from 90 to 100 having outstanding quality, 85 to 89.99 having excellent quality, 80 to 84.99 having very good quality and below 80 having below specialty quality. The lower scores also indicated increased PTD.

**Table 1:** Coffee IDs and taste scores

Bag ID	Taste score
SG 1359	81–82
SG 1447	81–83
SG 1507	81–83
SG 1516	81–83
SG 1405	81–82
SG 1550	82
SG 1452	82–83
SG 1454	82–83
SG 1420	72
SG 1512	77–80
SG 1347	79
SG 1386	79–80
SG 1366	79–80
SG 1474	69–80
SG 1505	79–81
SG 1441	79–82
SG 1416	60
SG 1419	60
SG 1422	60
SG 1466	60
SG 1464	60
SG 1465	60–72
SG 1556	62.5
SG 1529	68

### DNA isolation

Raw coffee bean samples were ground separately to fine powder at room temperature by processing them for 210 s in a Retsch MM300 grinder (90 s, 25 oscillations per second) using a 35-ml stainless-steel grinding jar (Retsch, Haan, Germany) and 20-mm stainless steel balls. Eight raw coffee beans from each subsample, weighed about 1.3–1.5 g, were randomly selected for the procedure. Microbial DNA was extracted from the fine coffee powders (200 mg) using the PowerSoil DNA Isolation Kit (MoBio Laboratories, Carlsbad, CA, USA) with a 60 s beat-beating step that used a Mini-Beadbeater-16 (BioSpec Products, Inc., Bartlesville, OK), and following the manufacturer's protocol except the DNAs were eluted in 1/10 EB buffer (Qiagen, Valencia, CA).

### Illumina fungal rRNA ITS library construction and sequencing

Illumina fungal rRNA ITS libraries were constructed as follows. PCRs were performed using a DNA Engine thermal cycler (BIO-RAD, Hercules, California) and 100-µl reactions containing: Phusion High-Fidelity DNA Polymerase Mix (New England Biolabs, Ipswich, MA, USA) supplemented with 500 μg/ml BSA, 1 mM MgCl_2_, 250 μM of each deoxynucleotide triphosphate (dNTP), 400 nM of each primer, and 4-μl of DNA template. The PCR primers gITS7 (GTGARTCATCGARTCTTTG) and ITS4 (TCCTCCGCTTATTGATATGC) targeted the ITS2 region of the ribosomal rRNA gene operon (Ihrmark et al. 2012; White et al. 1990), with the reverse primers including 7-base barcodes, and both primers including the Illumina sequences needed for cluster formation. Thermal cycling parameters were 94 °C for 5 min; 35 cycles of 94 °C for 20 s, 56 °C for 20 s, and 72 °C for 30 s; followed by 72 °C for 10 min. PCR products were purified using a QIAquick PCR Purification Kit (Qiagen) according to the manufacturer’s instructions. DNA sequencing (single-end 150 base) was performed using an Illumina MiSeq located at the Genomics Core Facility at the University of California, Riverside.

### Illumina fungal rRNA ITS sequence data processing and analyses

The UPARSE pipeline was used for de-multiplexing, length trimming, quality filtering, and operational taxonomic unit (OTU) picking using default parameters or recommended guidelines that were initially described in Edgar (2013) and which have been updated at https://www.drive5.com/usearch/manual/uparse_pipeline.html. Briefly, after demultiplexing and using the recommended 1.0 expected error threshold, sequences were kept at a uniform length of 151 bases. Sequences were then dereplicated and clustered into zero-radius OTUs (Z-OTUs) using the UNOISE3 algorithm (Edgar 2016), which detects and removes chimeric sequences. An OTU table was then generated using the otutab command. Taxonomic assignments of the fungal OTUs, or amplicon sequence variants (ASVs), were performed using the RDP Classifier version 2.12 (Wang et al. 2007), trained on the ver8_99_s_02.02.2019 release of the UNITE database (Kõljalg et al. 2013), and ASVs having nonfungal assignments were removed. Pearson correlation analyses were performed between fungal relative abundance values and coffee taste scores in R using the cor.test function and a log10, proportion-normalized OTU table, where the values were multiplied by 269,972 before the log10 operation such that the final non-zero values were greater than zero; the false discovery rate (FDR) method was also applied to these analyses (Benjamini and Hochberg 1995). Prism was used to make the correlation and taxa plots (GraphPad, La Jolla, CA). The fungal rRNA ITS sequences have been deposited in the National Center for Biotechnology Information (NCBI)’s Sequence Read Archive (SRA) under the BioProject Accession Number PRJNA640149.

## Results

**Fig. 1:**
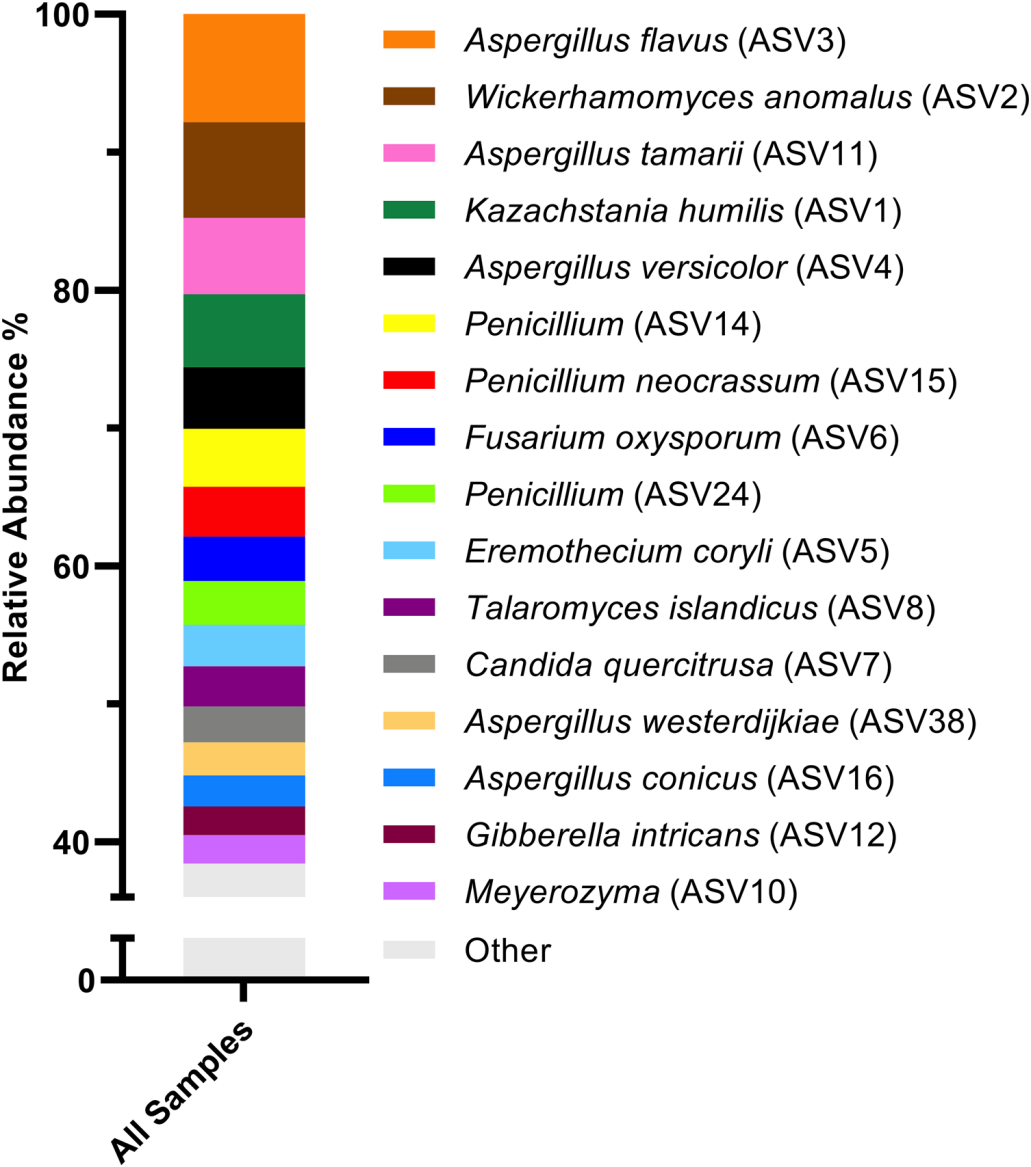
Abundant fungi in raw coffee beans from Rwanda. An Illumina-based sequence analysis of the fungal rRNA ITS region was used to determine the average relative abundances of ASVs (n = 24). ASV numbers are indicated in parentheses

An Illumina-based sequence analysis of the fungal rRNA ITS region was used to characterize the fungi associated with raw coffee beans from Rwanda (Fig. 1). The fungi with the highest relative abundances included several species of *Aspergillus*, *Wickerhamomyces anomalus*, *Kazachstania humilis*, and several *Penicillium* species, among others.

**Fig. 2:**
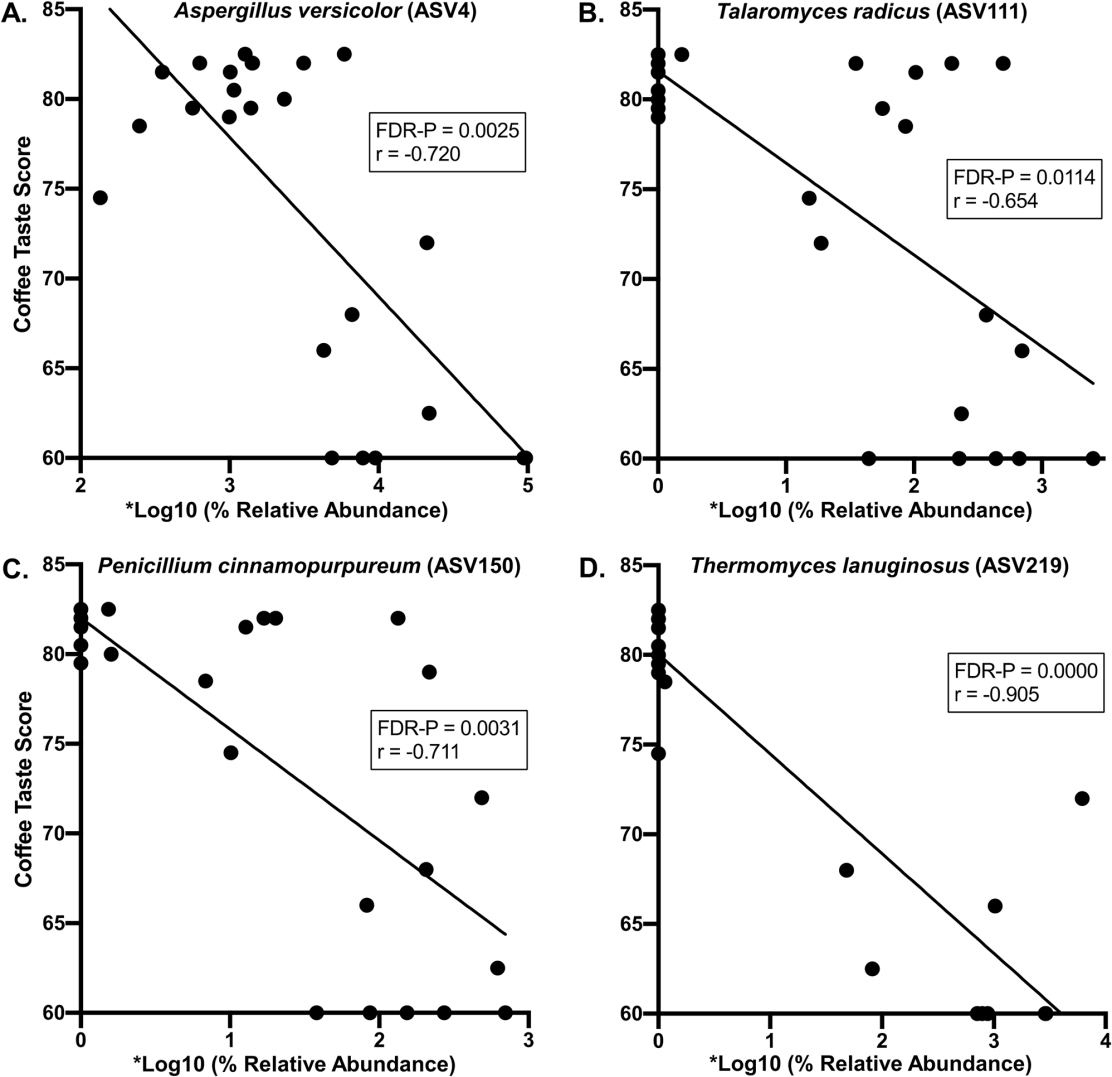
Abundant fungi that negatively correlated with coffee taste scores. Pearson correlation analyses were used to identify the abundant fungal ASVs that negatively correlated with coffee taste scores. Average ASV relative abundances were obtained using an Illumina-based sequence analysis of the fungal rRNA ITS region. *Relative abundance values were multiplied by 269,972 before the log10 operation such that the final non-zero values were greater than zero. ASV numbers are indicated in parentheses to the right of the taxa names. The average relative abundances of these fungi were: ASV4, 3.4%; ASV111, 1.5%; ASV 150, 1.3%; ASV219, 1.1%. Correlation coefficient (r) and probability value (P) are shown (n = 24)

**Fig. 3:**
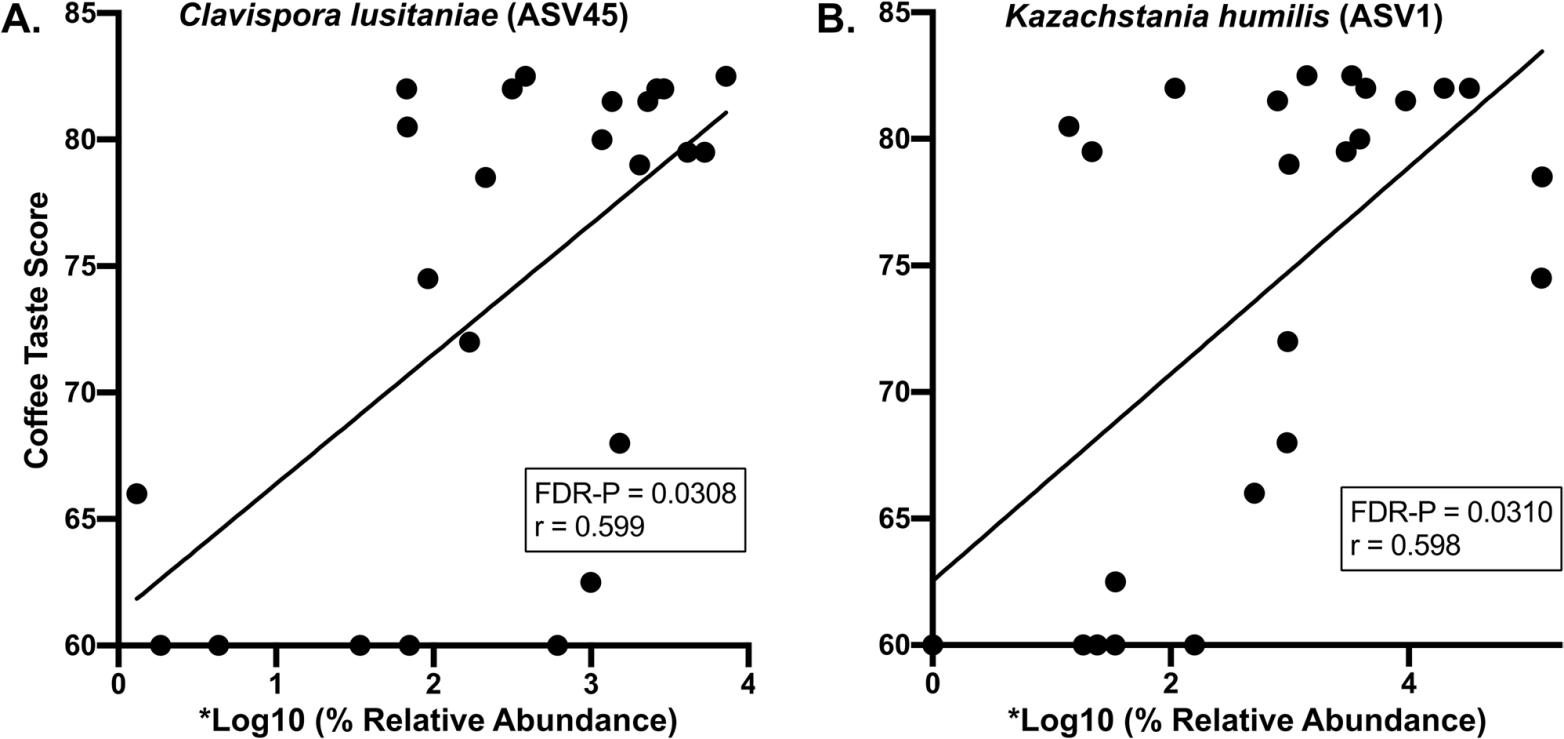
Abundant fungi that positively correlated with coffee taste scores. Pearson correlation analyses were used to identify the abundant fungal ASVs that positively correlated with coffee taste scores. Average ASV relative abundances were obtained using an Illumina-based sequence analysis of the fungal rRNA ITS region. *Relative abundance values were multiplied by 269,972 before the log10 operation such that the final non-zero values were greater than zero. ASV numbers are indicated in parentheses to the right of the taxa. The average relative abundances of these fungi were: ASV1, 2.8%; ASV45, 2.5%. Correlation coefficient (r) and probability value (P) are shown (n = 24)

Four fungi with high relative abundances exhibited negative correlations with coffee taste scores. They were *Aspergillus versicolor, Penicillium cinnamopurpureum, Talaromyces radicus,* and *Thermomyces lanuginosus* (Fig. 2). Their average relative abundance values ranged from 1.1% to 3.4%. Three of these fungi also exhibited density dependent patterns (Fig. 2A–C). Here, most of the samples typically belonged to one of two groups – one with lower relative abundance values and higher taste scores and the other with higher relative abundance values and lower taste scores. Two fungi with high relative abundances exhibited positive correlations with coffee taste scores. They were *Clavispora lusitaniae* and *Kazachstania humilis* (Fig. 3A, B), and their average relative abundance values were 2.8 and 2.5%, respectively.

## Discussion

Some of the most abundant fungi identified in our study were also identified in prior reports describing coffee bean microbes. A study by De Bruyn et al. (2017) in South America used high-throughput amplicon sequencing to examine the changes in the coffee bean microbiome throughout the post-harvest process from both the wet and dry methods (De Bruyn et al. 2017). The three most abundant fungi from that study were *Pichia kluyveri/fermentas, Starmerella bacillaris*, and *Candida quercitrusa*. *Candida quercitrusa* had the fourth highest relative abundance in our data, and it was the only abundant species our study had in common with the De Bruyn et al. (2017) study. Other genera found in both our study and the De Bruyn study were *Fusarium* and *Meyerozyma*. An investigation by Viegas et al. used culture-based methods to identify fungi on coffee beans from around the world (Viegas et al. 2017). Some of the most abundant fungi detected in that study were *Aspergillus* section Nigri, *Aspergillus* section Cirumdati, *Aspergillus* section Versicolor, and some *Penicillium* species. In a study in Brazil, Liardon et al. found *Aspergillus versicolor* on the surface of beans that had an unpalatable flavor, and a *Fusarium* sp. on the inside of these beans (Liardon et al. 1990). Several of the fungi identified in both of these investigations were also found in our study including *A. versicolor*, *A. nigri*, *P. neocrassum*, and *F. oxysporum* (Fig. 1, and not shown). In a study that used Illumina amplicon sequencing to examine the fungi associated with Columbian coffee, *Pichia nakasei*, *Dipodascus tretrasporeus*, and a *Candida* sp. were the most abundant fungi (de Oliveira Junqueira et al. 2019); however, in our study, the first two fungi were not detected and *Candida* spp. were found in lower relative abundances. Finally, a culture-dependent study of Brazilian coffee identified three fungi that were abundant in our study: *Aspergillus flavis*, *Aspergillus niger,* and a *Penicillium* sp. (Silva et al. 2008). Although our study did not examine bacteria, prior studies have found *Leuconostoc* spp., a *Lactococcus* sp. and a *Weissella* sp. to be abundant organisms during the fermentation process (De Bruyn et al. 2017). A cohort of lactic acid bacteria including a *Leuconostoc sp.*, a *Lactococcus sp.* and a *Lactobacillus sp.* dominated the later stages of the wet processing method.

In our findings, the negative correlations are consistent with organisms that might be causing PTD. A review of the literature found that *A. versicolor* (Fig. 2A) is a filamentous fungus that is associated with spoiled foods, including a taste aberration in Brazilian coffee, where this fungus was detected on the surface of the beans (Liardon et al. 1990). This fungus also produces volatiles associated with the pungent smell of mold in houses (Bjurman and Kristensson 1992). *Talaromyces radicus* (also *Penicillium radicum*) (Fig. 2B) produces high amounts of flavonoids, alkaloids, phenols, saponins, tannins and organic acids (Begum and Tamilselvi 2019). These compounds are described as having bitter flavors (Drewnowski and Gomez-Carneros 2000; Roland et al. 2013), except for saponins and organic acids, which have acrid (Drewnowski and Gomez-Carneros 2000) and sour flavors (Siebert 1999), respectively. *Penicillium cinnamopurpureum* (also *Eupenicillium cinnamopurpureum*) (Fig. 2C) is associated with bitter tasting cocoa beans (Rahmadi and Fleet 2008). We could not find any prior reports of *Thermomyces lanuginosus* (Fig. 2D) being associated with off-flavors or smells.

On the other hand, the positive correlations are also consistent with organisms that might be inhibiting PTD. A review of the literature found that *Clavispora lusitaniae* has antifungal properties. Accordingly, it has been shown to be an effective biological control agent against both *Penicillium digitatum*, which causes lemon fruit decay (Perez et al. 2019), and against *Penicillium roqueforti*, which causes spoilage of wheat grain (Lillbro 2005). *Kazachstania humilis* (also *Candida humilis*) is a yeast found in commercial sourdough bread production that improves its flavor (Carbonetto et al. 2020).

Based on our findings, we hypothesize that some of the negative and positive correlating fungi described in this study are organisms that are causing or inhibiting PTD, respectively. Future studies will employ Koch's postulates experimentation to test these hypotheses. We also posit that the density dependent correlation patterns shown in Fig. 2A–C are due to quorum sensing by these fungi. Quorum sensing is characterized by the production of specific metabolites when the population densities of an organism reaches a certain threshold. These metabolites mediate synchronized expression or repression of genes that perform a variety of functions such as biofilm formation, antifungal production, and antibiotic production, among others (Padder et al. 2018). Furthermore, quorum sensing has been shown to play a role in preventing food spoilage (Prathyusha et al. 2019). Although quorum sensing has been mostly studied in bacteria, it is also well-studied in certain fungal genera such as *Aspergillus*, *Candida* and *Saccharomyces* (Padder et al. 2018). In addition, because Antestia bugs are positively associated with PTD (Bigirimana et al. 2018), we hypothesize that they are one of the vectors transmitting the PTD-causing fungi. Finally, given that there are no reports linking PTD to obvious microbial infections of raw coffee beans, we posit that the causative fungi inhabit the interior of coffee beans. This latter hypothesis is supported by literature describing our negatively correlating fungal genera and species (Fig. 2) as plant endophytes (Abdelwahab et al. 2018; Ali et al. 2019; Begum and Tamilselvi 2019; Vega et al. 2006).

## Conclusions

In our study, four fungi had high relative abundances and negatively correlated with coffee taste scores, including *Aspergillus versicolor, Penicillium cinnamopurpureum, Talaromyces radicus,* and *Thermomyces lanuginosus*. In addition, *Clavispora lusitaniae* and *Kazachstania humilis* were found in high relative abundances and positively correlated with coffee taste scores. An analysis of the literature also determined that the negatively correlating fungi are consistent with organisms that might be causing PTD, and the positively correlating fungi are consistent with organisms that might be inhibiting the organisms that cause PTD. We therefore propose a hypothesis for PTD causation: the density dependent correlation patterns of the fungi and potato taste scores observed in this study came from fungi inhabiting the interior of coffee beans that are either contributing to or inhibiting PTD, and that involve quorum sensing mechanisms, and which are vectored by Antestia bugs.

## Data Availability

The datasets generated and analyzed during the current study are available in the National Center for Biotechnology Information (NCBI)’s Sequence Read Archive (SRA) under the BioProject Accession Number PRJNA640149.

## Abbreviations

PTD: Potato taste defect
OTU: Operational taxonomic unit
ASV: Amplicon sequence variants
